# Stabilization of circulating tumor cells in blood using a collection device with a preservative reagent

**DOI:** 10.1186/1475-2867-14-23

**Published:** 2014-03-07

**Authors:** Jianbing Qin, Jodi R Alt, Bradford A Hunsley, Thomas L Williams, M Rohan Fernando

**Affiliations:** 1 R&D Division, Streck, Inc., 7002 S 109 Street, La Vista, NE 68128, USA; 2 Methodist Hospital Laboratory, 8303 Dodge Street, Omaha, NE 68114, USA

**Keywords:** Circulating tumor cells, Blood collection devices, Clinical laboratory techniques

## Abstract

**Background:**

The enumeration and characterization of circulating tumor cells (CTCs) in the blood of cancer patients is useful for cancer prognostic and treatment monitoring purposes. The number of CTCs present in patient blood is very low; thus, robust technologies have been developed to enumerate and characterize CTCs in patient blood samples. One of the challenges to the clinical utility of CTCs is their inherent fragility, which makes these cells very unstable during transportation and storage of blood samples. In this study we investigated Cell-Free DNA BCT™ (BCT), a blood collection device, which stabilizes blood cells in a blood sample at room temperature (RT) for its ability to stabilize CTCs at RT for an extended period of time.

**Methods:**

Blood was drawn from each donor into K_3_EDTA tube, CellSave tube and BCT. Samples were then spiked with breast cancer cells (MCF-7), transported and stored at RT. Spiked cancer cells were counted using the Veridex CellSearch™ system on days 1 and 4. The effect of storage on the stability of proteins and nucleic acids in the spiked cells isolated from K_3_EDTA tube and BCT was determined using fluorescence staining and confocal laser scanning microscopy.

**Results:**

MCF-7 cell recovery significantly dropped when transported and stored in K_3_EDTA tubes. However, in blood collected into CellSave tubes and BCTs, the MCF-7 cell count was stable up to 4 days at RT. Epithelial cell adhesion molecule (EpCAM) and cytokeratin (CK) in MCF-7 cells isolated from BCTs was stable at RT for up to 4 days, whereas in MCF-7 cells isolated from K_3_EDTA blood showed reduced EpCAM and CK protein expression. Similarly, BCTs stabilized c-fos and cyclin D1 mRNAs as compared to K_3_EDTA tubes.

**Conclusion:**

Cell-Free DNA™ BCT blood collection device preserves and stabilizes CTCs in blood samples for at least 4 days at RT. This technology may facilitate the development of new non-invasive diagnostic and prognostic methodologies for CTC enumeration as well as characterization.

## Background

In the peripheral blood of patients with solid tumors of epithelial origin, some circulating cells have been found that have characteristics of tumor cells [1]. These cells that are present in the bloodstream of cancer patients, known as circulating tumor cells (CTCs), are thought to play an important role in cancer metastasis by breaking loose from a solid tumor, entering the circulation, and then migrating to distant organs to develop secondary tumors. The presence of CTCs in blood has been known for more than a century [2]. However, the clinical utility of CTCs was first shown in patients with metastatic breast cancer by Cristofanilli and colleagues [3]. The patient survival prognosis was more favorable with the identification of < 5 CTCs per 7.5 mL blood. CTCs are detectable in the blood of patients with metastatic cancer using different technologies [4]. Since CTCs are rare they need to be enriched from patient blood for accurate enumeration and characterization [5,6]. Most of the CTC enrichment and identification assays available today are based on enrichment with anti-EpCAM antibodies and subsequent identification using anti-CK antibodies [7,8]. An example is the CellSearch™ instrument system, a clinically validated system cleared by the USA Food and Drug Administration for isolation and enumeration of CTCs in blood of patients with metastatic breast, prostate and colorectal cancer [3,9].

There is a growing interest in the use of CTCs in non-invasive diagnosis, prognosis and monitoring of treatment regimens. The low abundance of the CTCs and their fragile nature may introduce variability in the evaluation of CTCs using different assay platforms. This fragile nature of CTCs arises due to the apoptosis of CTCs which begins after separation from the tumor of origin and after removal of blood from patient [9-11]. Therefore, it is necessary to address several pre-analytical issues that arise during the time between blood draw and CTC enrichment and characterization in order to effectively preserve CTCs for analysis. These include delays in blood processing, blood storage temperature, and agitation of the sample during transport and shipment of blood. Such conditions may affect the integrity of already fragile CTCs causing accurate enumeration and characterization of CTCs difficult. As a result, it is important to consider the type of blood collection device and post-phlebotomy conditions while working with CTC samples. Previous studies have shown that blood collection devices with cellular preservatives are capable of stabilizing CTCs for up to 96 hours [11-13].

Cell-Free DNA BCT™ is a blood collection device with a formaldehyde free stabilization reagent [14] that preserve cell-free DNA in a blood sample for up to 14 days at RT [15,16]. It does so by stabilizing nucleated blood cells in blood and preventing cellular DNA release into plasma [17]. This study was designed to investigate the effectiveness of this blood collection device for the stabilization of CTCs in blood sample for an extended period of time at RT.

## Results

### Recovery of spiked MCF-7 cells in blood

**Figure 1:**
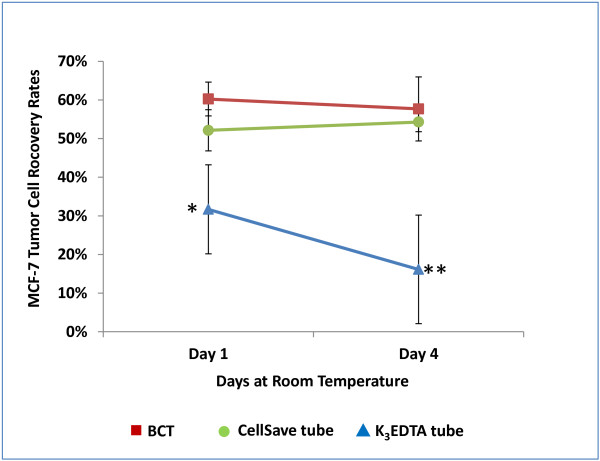
**Recovery of spiked MCF-7 cells in blood.** Normal donor blood was drawn into K_3_EDTA tubes, CellSave tubes and BCTs and a known number of breast tumor cells (MCF-7) were spiked. The whole blood samples were analyzed on CellSearch system to determine recovery of spiked MCF-7 cells at indicated time points. The tumor cell recovery from BCTs (red square symbol) and CellSave tubes (green circle symbol) was stable and much higher than from K_3_EDTA (blue triangle symbol) tubes after the blood samples were transported and stored at RT for 4 days. Samples drawn into K_3_EDTA tubes that were transported and stored at RT showed a statistically significant decrease in CTC count when compared to samples transported in the BCT and CellSave (*P < 0.001, **P < 0.0003). Error bars indicate standard deviation, n = 7.

Experiments were designed to determine the ability of BCTs to stabilize CTCs during blood sample storage and transportation compared to standard K_3_EDTA and CellSave blood collection tubes. Parallel blood samples drawn into K_3_EDTA, CellSave and BCTs spiked with MCF-7 cells were analyzed using CellSearch system for spiked tumor cell recovery. As shown in Figure 1, BCTs and CellSave tubes demonstrated stable percentage recovery of the tumor cells at RT for up to 4 days. In BCTs, at day 1 60% (Standard deviation (SD) = 4%, coefficient of variation (CV) = 7.3%) of spiked MCF-7 cells were recovered and at day 4 it was 58% (SD = 8%, CV = 14.3%). Similarly, in CellSave tubes at day 1 52% (Standard deviation (SD) = 5%, coefficient of variation (CV) = 10.2%) of spiked MCF-7 cells were recovered and at day 4 it was 54% (SD = 2%, CV = 4.6%). In contrast, K_3_EDTA tubes failed to preserve CTCs resulting in a much lower recovery rates for both day 1 and 4 as compared to BCTs. In K_3_EDTA tubes, at day 1, recovery rate was 32% (SD = 12%, CV = 36.3%) of the spiked MCF-7 cells and at day 4 it was 16% (SD = 14%, CV = 87%).

### Stability of EpCAM and CK proteins by immunofluorescence

**Figure 2:**
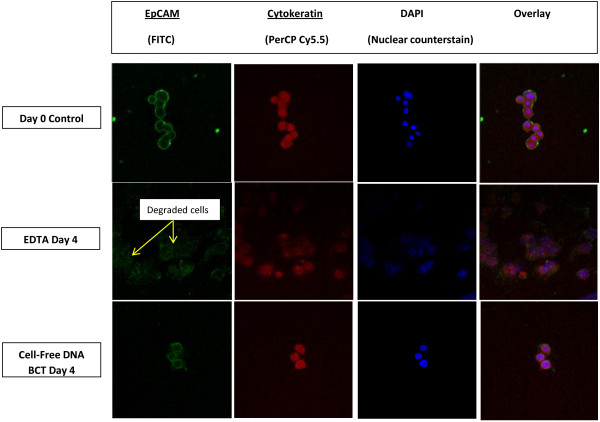
**Comparison of tumor cell EpCAM and CK protein stability in BCTs and K**_**3 **_**EDTA tubes.** Blood was drawn into K_3_EDTA tubes and BCTs and plasma was isolated. MCF-7 cell were spiked into plasma and stored at RT. Cytospin samples were prepared at indicated time points and expressions of EpCAM and CK proteins were determined using the standard immunofluorescence cell staining protocol as described in the “Materials and Methods” section. Tumor cell proteins, EpCAM and CK, cell nucleus and nuclear content were stable in BCTs at RT for 4 days. However, tumor cells incubated in K_3_EDTA plasma showed degrading EpCAM and CK proteins, nucleus and nuclear content upon storage at RT for 4 days. Original magnification x 40. Representative staining results from 3 independent experiments.

Figure 2 illustrates the effects of RT storage on the stability of tumor-associated trans-membrane protein EpCAM and cytoskeleton protein CK of MCF-7 tumor cells spiked into blood plasma. The EpCAM protein (green fluorescence) was stable up to 4 days at RT in BCTs whereas in K_3_EDTA tubes this membrane protein was partially degraded by day 4 as evidenced from the weak and diffused fluorescence signal for EpCAM cell membrane protein. The CK protein signal (red fluorescence) appears to be unchanged in BCTs up to 4 days. However, the reduced fluorescence intensity for this protein in spiked MCF-7 cells recovered from K_3_EDTA tubes suggests it was less stable than the BCT samples. When cells were stained with DAPI, the nucleus and nuclear content appear unchanged in cells recovered from BCTs but not cells recovered from K_3_EDTA tubes after 4 days of RT storage.

### Stability of mRNA molecules using molecular beacons

**Figure 3:**
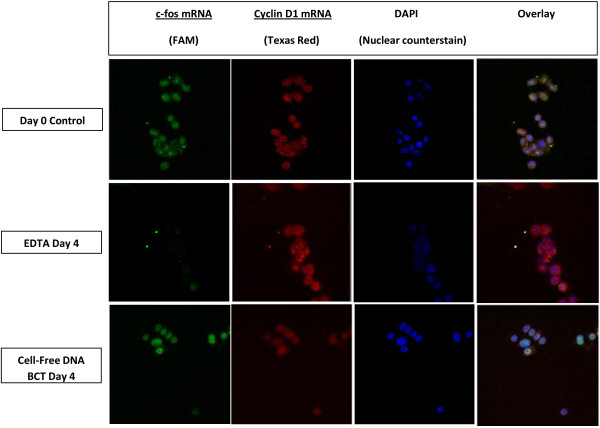
**Comparison of tumor cell mRNA stability in BCTs and K**_**3 **_**EDTA tubes.** MCF-7 cells spiked into K_3_EDTA and BCT plasma were isolated at indicated time points and cytospin samples were prepared as described in the “Materials and Methods” section. MCF-7 cells on cytospin were stained with fluorescent-labeled molecular beacons for c-fos (green fluorescence) and cyclin D1 (red fluorescence) mRNAs. The confocal microscopic images show that c-fos and cyclin D1 mRNA levels did not change in tumor cells incubated in plasma from BCT at RT for 4 days. However, tumor cells incubated in K_3_EDTA plasma for 4 days at RT showed a decrease in fluorescence for c-fos mRNA compared to day 0 fluorescence but cyclin D1 mRNA showed a slight increase upon storage at RT for 4 days. Original magnification × 40. Representative staining results from 3 independent experiments.

Experiments were performed to study the stability of mRNA in the spiked MCF-7 cells recovered from BCTs and K_3_EDTA tubes. Slides of the recovered MCF-7 cells were prepared as described above. To detect mRNA in situ, fluorescent-labeled molecular beacons and a scanning confocal microscope were used. As shown in Figure 3, c-fos mRNA (green fluorescence) and cyclin D1 mRNA (red fluorescence) showed similar intensity in cells on day 0 and day 4 when stored in BCTs. However in K_3_EDTA samples c-fos mRNA signal was reduced after 4 days of storage at RT, suggesting that c-fos mRNA expression was degraded or downregulated. There was slight increase in cyclin D1 mRNA level in MCF-7 cells recovered from K_3_EDTA tube after 4 days of incubation at RT.

## Discussion

The presence of CTCs in patients with cancer has been known for over a century [2]. However, utilization of these rare cells in cancer diagnosis and prognosis was not feasible since methodologies to detect, isolate and characterize CTCs have not been developed until recently. With the development of robust methodologies to enrich, isolate and characterize CTCs in different types of solid organ cancers, several clinical studies have now been conducted to investigate the possible use of CTCs in cancer diagnosis and prognosis [3,18]. Assays that enumerate CTCs using the CellSearch system have been developed as an aid to monitor patients with metastatic breast, colorectal, and prostate cancers [3,19,20]. The potential usefulness of CTC enumeration has also been demonstrated with melanoma, urothelial, and lung cancer [21-23].

Factors that limit the utility of CTCs in cancer diagnosis and prognosis are the low abundance and the fragility of the CTCs. These factors may introduce variability in the evaluation of CTCs using different assay platforms [24]. Transportation of blood samples from the site of phlebotomy to another facility is commonly required for CTC enumeration and characterization. During post-phlebotomy blood sample transportation and storage, fragile CTCs may degrade further compromising the accuracy of CTC enumeration and characterization [11-13]. In our study, we have minimized CTC degradation with a novel cell stabilizing reagent contained within a blood collection tube.

In our experiment described in Figure 1, we modeled transportation as well as storage effects on CTCs in blood samples. We spiked 2000 MCF-7 cells into blood contained in standard K_3_EDTA and CellSave tubes or the novel BCTs. These samples were then shipped from Omaha, NE to Maryville, TN for analysis by the CellSearch system. Our CTC recovery study conducted using spiked MCF-7 cells provides evidence that BCTs are able to preserve CTCs during transportation and storage at RT for up to 4 days, similar to CellSave tube which is an integral part of CellSearch System. Previous studies using the CellSearch have shown that the recovery rate for MCF-7 cells is between 62 – 89% [25]. Our results show that BCTs, post shipping day 1 and day 4, had recovery rates of 61% and 57%, respectively. There was no statistically significant difference between these two values indicating that CTCs are stable in BCTs for 4 days after shipping at RT. However, in K_3_EDTA tubes, CTC recovery rate was very low compared to BCTs. The K_3_EDTA tubes from the same study showed day 1 and day 4 recovery rates of 32% and 16%. There was a statistically significant decrease in CTC recovery in K_3_EDTA tube between day 1 and day 4 compared to BCTs.

As shown in Figure 2, immunofluorescence staining of recovered CTCs for EpCAM and CK showed stability of these proteins from BCTs after 4 days of RT storage. However, cells recovered from K_3_EDTA tubes showed degrading EpCAM and CK proteins after 4 days under the same conditions. DAPI staining of cells showed stable nucleus and nuclear content in CTCs recovered from BCTs whereas CTCs recovered from K_3_EDTA tubes showed degrading nucleus and nuclear content. Our molecular beacon study (Figure 3) shows that both c-fos and cycling D1 mRNA are stable in BCTs at RT for up to 4 days. The stability of both c-fos and cyclin D1 mRNAs in BCT may results from the stabilization of tumor cells by the stabilizing reagent present in the BCT.

However, CTCs incubated in K_3_EDTA plasma for 4 days at RT showed almost complete degradation of c-fos mRNA whereas cyclin D1 mRNA level was slightly increased. We speculate that in degrading MCF-7 cells, c-fos gene may be down-regulated and cyclin D1 gene may be up-regulated.

Analysis of the stabilizing reagent present in BCT device by ^13^C-NMR has shown that the reagent is free of formaldehyde [14]. Aldehyde based chemicals traditionally used in cell stabilization, such as formaldehyde and glutaraldehyde, are known to damage DNA and RNA by causing chemical modifications in nucleic acids [26]. Application of such aldehyde based chemicals for CTC stabilization may cause problems for CTC characterization studies. Cell stabilizing reagent present in BCTs has an advantage over aldehyde based stabilizing agents because it has no negative effect on DNA amplification by PCR [27].

## Conclusions

In this study, we have modeled various circumstances that could alter CTC detection on an FDA cleared instrument running assays that are designed to be helpful in cancer diagnosis, prognosis and the monitoring of patient response to treatments. The modeling of post-phlebotomy has shown that BCT provides preservation and stabilization of CTCs in blood samples for up to 4 days at RT while a standard K_3_EDTA tube does not. By using BCT for future studies, it could facilitate the development of new non-invasive diagnostic and prognostic methodologies for CTC enumeration as well as characterization.

## Materials and methods

### Blood sample collection

This study was approved by the institutional review board of the Methodist Hospital, Omaha, NE, USA, and informed consent was obtained from all donors prior to blood draw. Blood specimens were collected from apparently healthy adult donors by standard phlebotomy techniques.

### Cell culture

Breast cancer cell line, MCF-7, was obtained from American Type Culture Collection (Rockville, MD, USA) and routinely passaged in Eagle’s MEM medium containing 10% fetal bovine serum at 37 C in humidified atmosphere of 5% CO_2_.

### Recovery of spiked MCF-7 cells in blood

For MCF-7 cell spiking experiments, blood from each donor (7 donors in total) was drawn into two 10 mL K_3_EDTA tubes (BD Vacutainer®, Becton Dickinson, Franklin Lakes, NJ, USA), two 10 mL CellSave tubes (Veridex, North Raritan, NJ, USA) and two 10 mL BCTs (Streck Inc., Omaha, NE, USA). A known number of MCF-7 cells (2000 cells/10 mL of whole blood) were then spiked into each tube and the samples were mixed immediately by inverting 10 times each. All samples were shipped at ambient temperature to Geneuity Clinical Research Services (Maryville, TN, USA). The samples were analyzed on days 1 and 4, post phlebotomy, on the Veridex CellSearch system in order to count the recovery rate of the MCF-7 cells. Blood samples were maintained at RT during the entire process.

### Detection of EpCAM and CK by immunofluorescence cell staining

Blood was drawn from each donor into one 10 mL K_3_EDTA tube and one 10 mL BCT. Plasma was separated from blood within 2 h post collection. To separate plasma, blood samples were centrifuged at 300 × *g* for 20 min at RT. The upper plasma layer was carefully removed without disturbing the buffy coat and transferred to a fresh tube and centrifuged again at 5000 × *g* for 10 min. MCF-7 cells (≈ 2,000 cells/4 - 5 mL of plasma) were spiked into the cell-free plasma and stored at RT. On days 0 and 4, MCF-7 cells were centrifuged at 500 rpm for 7 min on glass slides using Shandon Cytospin® 3 cytocentrifuge. Slides were dried and immunostained with a primary antibody cocktail containing a mouse anti-EpCAM antibody (VU-1D9, #sc-51681, 1:100) and a mouse anti-CK antibody (T-13, #sc-241376, 1:100). After 1 h of incubation, slides were washed twice with PBS and probed with fluorescent labeled secondary antibodies for mouse anti-EpCAM (donkey anti-mouse IgG-FITC, #sc-2099, 1:200) and mouse anti-CK (donkey anti-goat IgG-PerCP-Cy5.5, #sc-45102, 1:200) antibodies for 1 h. After again washing slides two times with PBS, coverslips were mounted onto slides with UltraCruz™ mounting medium (#sc-24941) containing 4′, 6-diamidino-2-phenylindole (DAPI) to counterstain cell nuclei. All antibodies and mounting medium were purchased from Santa Cruz Biotechnology, Inc. (Dallas, TX, USA) and manufacturer’s protocol was followed. Fluorescent images were obtained using Zeiss LSM 510 META NLO laser scanning confocal microscope (Oberkochen, Germany).

### In situ detection of mRNA using molecular beacons

Cytospin slides of MCF-7 cells in cell-free plasma were prepared as described above. Cells on the slides were fixed and permeabilized with ice cold methanol (-10°C) for 10 min. After air drying, slides were stained with a mixture of 200 nmol/L of fluorescent-tagged molecular beacons targeting c-fos or cyclin D1 mRNAs in Opti-MEM (Invitrogen) at 37°C for 1 h. Slides were washed, countstained with DAPI and examined using a confocal microscope. The sequences of molecular beacons are 5′-6-FAM-CGACCTCTAGTTGGTCTGTCTCCGCGGTGG-Dabcyl-3′ for c-fos and 5′-Texas-Red-TGGAGTTGTCGGTGTAGACTCCA-Dabcyl-3′ for cyclin D1, which were purchased from Eurofins MWG Operon (Huntsville, AL).

### Statistical analysis

Statistical analysis was carried out using Microsoft Excel for Office 2007. Analysis was performed using paired, two tailed Student’s *t*-test and p < 0.05 was considered statistically significant.

## Abbreviations

CTC: Circulating tumor cell; BCT: Cell-Free DNA™ BCT; RT: Room temperature; EpCAM: Epithelial cell adhesion molecule; CK: Cytokeratin.

## Competing interests

TW declares that no conflicts of interest exist. All other authors are full time employees of Streck Inc.

## Authors’ contributions

JQ participated in the experimental design, performed the laboratory work, carried out data and statistical analyses, interpreted the results, prepared and revised the manuscript. JRA participated in experiment optimization and revised the manuscript. BAH coordinated the CellSearch study and revised the manuscript. TW was responsible for IRB of blood sample collection, reviewed the manuscript. MRF conceived the study, participated in its design and the laboratory work, interpreted the results, prepared and revised the manuscript. All authors have read and approved the final manuscript.
